# Repetitive Transcranial Magnetic Stimulation in the Treatment of Alzheimer’s Disease and Other Dementias

**DOI:** 10.3390/healthcare9080949

**Published:** 2021-07-28

**Authors:** Athina-Maria Aloizou, Georgia Pateraki, Konstantinos Anargyros, Vasileios Siokas, Christos Bakirtzis, Markos Sgantzos, Lambros Messinis, Grigorios Nasios, Eleni Peristeri, Dimitrios P. Bogdanos, Triantafyllos K. Doskas, Georgios Tzeferakos, Efthimios Dardiotis

**Affiliations:** 1 Department of Neurology, Laboratory of Neurogenetics, University Hospital of Larissa, Faculty of Medicine, School of Health Sciences, University of Thessaly Biopolis, Mezourlo Hill, 41100 Larissa, Greece; athena_aloi@yahoo.gr (A.-M.A.); georginapat08@gmail.com (G.P.); konanarg@gmail.com (K.A.); vsiokas@med.uth.gr (V.S.); sgantzosmarkos@gmail.com (M.S.); eperiste@uth.gr (E.P.); 2 Multiple Sclerosis Center, B’Department of Neurology, AHEPA University Hospital, Aristotle University of Thessaloniki, 54636 Thessaloniki, Greece; bakirtzischristos@yahoo.gr; 3 Neuropsychology Section, Departments of Neurology and Psychiatry, University Hospital of Patras and University of Patras, Medical School, 26504 Patras, Greece; lmessinis@upatras.gr; 4 Department of Speech and Language Therapy, School of Health Sciences, University of Ioannina, 45500 Ioannina, Greece; grigoriosnasios@gmail.com; 5 Department of Rheumatology and Clinical Immunology, University General Hospital of Larissa, Faculty of Medicine, School of Health Sciences, University of Thessaly, Viopolis, 40500 Larissa, Greece; bogdanos@uth.gr; 6 Department of Neurology, Athens Naval Hospital, Deinokratous 70, 11521 Athens, Greece; doskastr@gmail.com; 7 Association for Regional Development and Mental Health (EPAPSY), 15124 Marousi, Greece; g.tzeferakos@epapsy.gr

**Keywords:** rTMS, dementia, Alzheimer’s disease, magnetic stimulation, non-pharmacological treatments

## Abstract

Dementia is a debilitating impairment of cognitive functions that affects millions of people worldwide. There are several diseases belonging to the dementia spectrum, most prominently Alzheimer’s disease (AD), vascular dementia (VD), Lewy body dementia (LBD) and frontotemporal dementia (FTD). Repetitive transcranial magnetic stimulation (rTMS) is a safe, non-invasive form of brain stimulation that utilizes a magnetic coil to generate an electrical field and induce numerous changes in the brain. It is considered efficacious for the treatment of various neuropsychiatric disorders. In this paper, we review the available studies involving rTMS in the treatment of these dementia types. The majority of studies have involved AD and shown beneficial effects, either as a standalone, or as an add-on to standard-of-care pharmacological treatment and cognitive training. The dorsolateral prefrontal cortex seems to hold a central position in the applied protocols, but several parameters still need to be defined. In addition, rTMS has shown potential in mild cognitive impairment as well. Regarding the remaining dementias, research is still at preliminary phases, and large, randomized studies are currently lacking.

## 1. Introduction

Dementia is a serious health issue around the globe, with a huge social and economic burden, since it affects a large proportion of an otherwise possibly healthy population that is steadily rendered incapable of self-care [1]. There are several dementia types or/and diseases that can cause dementia, such as Alzheimer’s disease (AD), vascular dementia (VD), Lewy body dementia (LBD) and frontotemporal dementia (FTD). Pharmacological treatments for these diseases have been more or less ineffective in halting disease progress and ameliorating symptoms, and as such, other non-pharmacological treatment options are now being explored [2].

Transcranial magnetic stimulation (TMS) was introduced as a non-invasive brain stimulation technique by Barker et al. [3]. In TMS, a magnetic coil over the skull generates a high-intensity pulse which can stimulate neurons, and the stimulation varies according to several technical parameters of the coil and the protocol applied [4]. Single and paired stimuli (single- and paired-pulse TMS) are usually used for physiological/pathophysiological studies, while a series of repetitive stimuli (repetitive TMS, rTMS) can induce alterations that persist in the brain. rTMS has found application in various therapeutic protocols and is being tested in several neurodegenerative diseases, with cognitive sequelae as well, such as multiple sclerosis [5].

rTMS induces changes and influences neuronal blood circulation, metabolism and excitability in the stimulated region and other regions connected to the stimuli focus [6,7,8]. Its effects can be either excitatory or inhibitory, depending on several rTMS protocol parameters, such as frequency, duration and intensity, as expressed by a percentage of the motor threshold (MT, active or resting, AMT/RMT). The main categorization is based on frequency, with two main rTMS types: low-frequency rTMS (LF-rTMS) (≤1 Hz), known to produce inhibitory results, and high-frequency rTMS (HF-rTMS) (>1 Hz), with excitatory results [9]. The reported aftereffects following the repetitive stimulation are considered to reflect synaptic modulations, based on the principles of long-term potentiation (LTP) and long-term depression (LTD), the balance of which is implicated in important cognitive functions, memory included [8,9].

The aim of this review is to summarize the available literature on the therapeutic application of rTMS in the most frequent dementia types and to discuss how the scientific community should proceed in future studies. Additionally, we present ongoing studies on the matter, whose results are expected in the future. To the best of our knowledge, this is the first review to cover the subject of rTMS in the entirety of the dementia subtypes.

## 2. Alzheimer’s Disease

**Figure 1:**
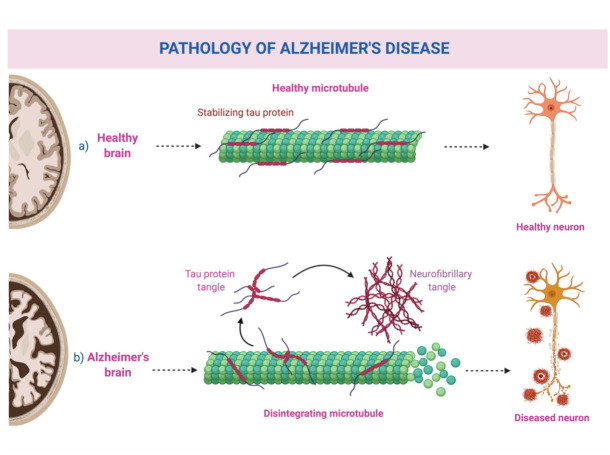
Schematic representation of Alzheimer’s disease pathology. The figure was prepared using a BioRender template under license (to DPB).

Alzheimer’s disease (AD) is the commonest form of dementia in older populations; in fact, almost 5% of people under 65 are affected, with this percentage rising considerably with age, reaching 40–50% in those aged 85 and over [2]. It is considered a neurodegenerative disease, with cognitive impairment, mainly regarding memory and orientation, and behavioral disorders being the most frequently reported symptoms [2]. A plethora of genetic [10,11] and environmental factors [12] have been implicated in its pathogenesis, with no definitive causative factor having been identified so far. Its pathological hallmarks are extracellular amyloid-beta plaques, also known as senile plaques, and neurofibrillary tangles from hyperphosphorylated tau protein [12,13]. A schematic representation can be seen in Figure 1.

To date, acetylcholinesterase inhibitors represent the main therapeutic options, but their efficacy in symptom alleviation and disease progress delay is limited [14,15]. As the average life expectancy rises, AD is expected to affect many more millions in the future. It is therefore imperative to find treatments that are actually effective in slowing and possibly reversing its processes, since pharmaceutical agents have proven more or less disappointing, and research has delved into novel pathways involving pathophysiological mechanisms such as endoplasmic reticulum (ER) stress [16]. Non-pharmacological approaches have steadily gained more ground as well, such as cognitive training [17]. In fact, an earlier meta-analysis reported that non-invasive techniques had a significant positive effect on cognitive outcomes [2] and are therefore a promising alternative.

rTMS in the context of dementia has been principally explored in AD, as it is thought to enhance synaptic plasticity, something that can be of utmost importance in preserving cognitive function. As shall be analyzed in detail below, the protocol designs have mostly involved the dorsolateral prefrontal cortex (DLPFC) and regions associated with specific cognitive functions, such as language, memory and attention. Additionally, study designs have been characterized as “online” or “offline” based on whether the stimulation was applied during the course of the cognitive tasks or not, with “online” designs seemingly producing stronger results [2]. Below, we have divided the relevant studies into those focusing on the DLPFC, those that combined rTMS and cognitive training, and those that applied stimulation over different areas. DLPFC studies are presented in Table 1, while studies involving cognitive training can be found in Table 2.

### 2.1. Stimulation of the Dorsolateral Prefrontal Cortex

The DLPFC has been targeted in a number of studies mainly assessing language in AD. Cotelli et al. (2006) assessed the effects of the left and right DLPFC HF-rTMS (20 Hz, 90% MT intensity) on picture naming during stimulation (“online”) [18]. They enrolled 15 anomic AD patients, and performed three blocks of naming tasks, one while they stimulated the left side, one for the right and one for sham. They reported that action/verb naming significantly improved during stimulation of both regions, a finding that was not reported for object/noun naming. The authors also claimed that, compared to other studies that showed improved scores only for left-sided stimulation in normal subjects, this bilateral effect in AD patients probably reflects compensating mechanisms that recruit right-sided networks to support naming and other cognitive functions. The same group, two years later (2008), published another “online” study on DLPFC HF-rTMS (same basic parameters) and naming, examining 24 AD patients with various degrees of cognitive decline [19]. Again, they reported improved action- (but not object-) naming after stimulation of both sides for subjects with mild cognitive decline. On the contrary, naming in both types improved in the individuals with moderate to severe decline. In 2011, the same researchers aimed to further examine this region’s effect on language performance and enrolled 10 AD patients, divided into two groups, one receiving real HF-rTMS (20 Hz, 100% MT intensity) stimulation for 4 weeks (5 days/week) over the left DLPFC, and one receiving sham for two weeks and then real stimulation for the subsequent two weeks [20]. They assessed the patients (“offline”) at two weeks, four weeks and twelve weeks after initiation, so the last follow-up session was 2 months after the stimulation protocol had ended. At two weeks, there was a significant improvement in auditory sentence comprehension in those receiving real rTMS, which persisted when assessed at follow-up. Other cognitive functions such as memory or other language abilities, naming included, did not show any significant effects, unlike their previous research; they attributed this difference to the different (online vs. offline) designs. The researchers also reported that no additional positive effects were noted from the additional two weeks of rTMS. The wealth of data provided by these three studies was hampered by the fact that all the published data originated from the same study group. One year later, Haffen et al. (2012) described a case of a 75-year-old man with AD, under AD treatment and an antidepressant agent, who received 2 weeks (10 sessions) of HF-rTMS (10 Hz, 100% MT intensity) over his left DLPFC [21]. When he was examined one month after the protocol had ended, he showed improvement in the majority of neuropsychological tests, especially in processing speed and episodic memory, while his environment reported that there was a notable improvement in his everyday activities. No adverse effects or new depressive episodes were noted.

Rutherford et al. (2015) conducted a pilot two-stage study on AD patients with either early or advanced disease [23]. The first stage was a double-blinded, crossover study on nine AD patients, who received 13 sessions of HF-rTMS (20 Hz, 90–100% RMT intensity) over the bilateral DLPFC in the span of 4 weeks. The second stage included blocks of two weeks with 10 real sessions every 3 months, as a follow-up, for six patients that also completed the first phase (min. 10 months, max. 19 months). Montreal Cognitive Assessment (MoCA), Alzheimer’s Disease Assessment Scale–Cognitive Subscale (ADAS–Cog) and Revised Memory and Behavior Checklist (RMBC) scores were used for cognitive assessment at baseline, 4 weeks after the last session of the first phase and at the follow-up sessions. Due to several methodological pitfalls, such as scheduling issues for the assessments, most of the results did not reach significance levels. However, an improvement in ADAS-Cog and RMBC was noted after real stimulation. Computerized cognitive exercises were also assessed in a subgroup of patients, where those receiving real training also seemed to perform better, with the scores regarding word–image association reaching the significance threshold. Additionally, patients in the early stages showed a greater overall responsiveness to the treatment, and when analyzed alone, their MoCA scores for the first weeks were significantly better when real was compared to sham. 

Wu et al. (2015) assessed the effectiveness of HF-rTMS (20 Hz, 80% MT intensity) over the left DLPFC on cognition and behavioral and psychological symptoms accompanying AD in a double-blinded study [24]. They randomized 54 patients with such symptoms to either active or sham stimulation for five days per week for four weeks, alongside their antipsychotic medication. Ultimately, 26 patients from each treatment branch completed the protocol. The patients were assessed by means of the Behavioral Pathology in Alzheimer’s Disease Rating Scale (BEHAVE-AD), ADAS-Cog and the Treatment Emergent Symptom Scale (TESS) before and after the 4-week protocol. Upon controlling for baseline performances, patients receiving active stimulation had significantly better (i.e., decreased) BEHAVE-AD scores, specifically regarding five of the seven subscore scales, namely activity disturbances, diurnal rhythm, aggressiveness, affective disturbances, anxieties and phobias. They further presented significant improvement in ADAS-Cog scores, compared to sham, in all of the assessed domains, namely language, praxis, memory and attention. Regarding behavioral and psychological symptoms, a higher proportion of patients in the active group showed improvement (73.1% vs. 41.7%). This study holds particular value due to its design, and the fact that it included a larger number of patients than other studies in the same field.

What can be easily deduced from these studies is that HF-rTMS is the method of choice for AD protocols, and that LF-rTMS studies are lacking, since LF-rTMS is not thought to produce beneficial results for these patients. In fact, earlier results suggest that LF-rTMS might even lead to deterioration [33]. To validate this notion, Ahmed et al. (2012) enrolled 45 mild to severe AD patients and divided them equally into three groups [22]. The first group received real HF-rTMS over the DLPFC bilaterally (20 Hz, 100% MT intensity), the second group received LF-rTMS (1 Hz, 100% MT), and the third received sham. The right DLPFC and then the left were stimulated, for one session per day for five consecutive days. The patients were assessed with the Mini Mental State Examination (MMSE), the Instrumental Daily Living Activity (IADL) scale and the Geriatric Depression Scale (GDS) before and after the whole intervention, and then after 1 and 3 months (“offline”). In all the assessment time-points after the intervention, the HF-rTMS group exhibited significantly better scores than the sham and the LF-rTMS groups, despite having no difference at baseline. As improvement persisted at 3 months, the results of this study suggest that rTMS may affect cognition in the long-term. It should be mentioned, however, that only patients with mild to moderate AD responded to treatment, since those with severe dementia did not show improvement in any of the treatment arms.

In this context, the matter of the right DLPFC needs to be discussed. The left DLPFC has been the main focus of the aforementioned studies, which either stimulated both cortices or solely the left. Studies have shown that the recruitment of the right DLPFC occurs in individuals with memory deficits [34], but whether this activation reflects effective compensatory mechanisms [35], or mechanisms with negative impact [36] remains contradictory. In this line of thought, Turrizziani et al. (2012) conducted a study involving 100 healthy young individuals and 8 MCI patients (more studies of this kind shall be analyzed in a section to follow) [37]. They applied verbal and non-verbal recognition tasks (episodic memory) in four sets of experiments, and the stimulation was applied before the recognition, as either LF-rTMS (1 Hz, 90% MT) or HF-rTMS in the form of intermittent theta burst stimulation (three pulses of 50 Hz, 80% AMT) and sham. In the first experiment set, 20 subjects were to receive LF-rTMS and sham over the left DLPFC, and 20 subjects over the right DLPFC, and then participate into a non-verbal recognition memory task. In the second set, the same design was maintained, with 40 different participants, but verbal recognition was tested instead. In the third experiment, ten participants received HF-rTMS over the left DLPFC and then received HF-rTMS over the right, and were tested on non-verbal recognition. Finally, on the fourth experiment, the MCI patients received LF-rTMS to the left and right DLPFC and sham, all in different sessions. They were also tested on non-verbal recognition. The results showed that LF-rTMS over the right and not the left DLPFC significantly improved test accuracy compared to sham in both verbal and non-verbal recognition, while HF-rTMS over the right DLPFC significantly decreased accuracy compared to sham. HF-rTMS over the left DLPFC did not improve test accuracy in the healthy subjects. Regarding the MCI patients, all eight showed non-verbal recognition improvement upon LF-rTMS over the right DLPFC, but not the left, like the first experiment in the healthy controls. This study showed that the activation of the right DLPFC probably negatively impacts memory processes, as showed by the improved test results upon inhibitory stimulation of the right DLPFC. The same research group further explored the effects of LF-rTMS over the right DLPFC in AD patients [25]. They conducted two experiments; in the first, 24 mild AD patients received LF-rTMS (1 Hz, 90% MT) and sham over one hemisphere before a non-verbal recognition test, and two weeks later the other hemisphere was stimulated and tested in a similar manner. In the second experiment, 14 AD patients were randomized to receive either real LF-rTMS over the right DLPFC or sham, for two weeks (5 days per week), and were then assessed at the end of the two weeks and two weeks after that (one month from the protocol initiation). They reported that in the first branch, real stimulation over the right DLPFC led to significant improvement in test accuracy compared to sham, while no difference between real and sham was noted for the left DLPFC. In the second branch, real stimulation was shown to improve performance after the two weeks, which was maintained when assessed at one month follow-up.

Taken together, these results suggest that HF-rTMS over the DLPFC might be beneficial regarding language and other cognitive functions for patients, possibly even in more advanced stages. They also highlight the fact that the brain employs several mechanisms to counterbalance impaired functions, which rTMS seems to affect. One theory to explain this effect is based on dopamine, since studies have shown that HF-rTMS over the DLPFC enhances dopamine production in areas such as the caudate nucleus [38]. Additionally, the effects of rTMS over this region seem to persist through time, albeit this being shown less consistently. However, caution must be exercised overall, as rTMS over the prefrontal cortex has been shown to inhibit other processes, such as memory [39], and the implication of the right DLPFC has yet to be elucidated Thus, its effects need to be assessed regarding all cognitive domains, in order to ensure that it does not aggravate other symptoms, and to define optimal parameters and stimulation targets.

### 2.2. Combination of HF-rTMS and Cognitive Training

Another line of mostly “offline” studies has studied the combined effects of rTMS and cognitive training, and has also included the DLPFC. These studies have mostly exploited the set structure of the NeuroAD™ protocol, as described below.

First Bentwich et al. (2011) enrolled eight mild to moderate AD patients (data for only seven of whom were included due to a withdrawal) and administered HF-rTMS (10 Hz, 90% MT intensity) with concomitant cognitive training for 6 weeks (5 days/week), followed by maintenance sessions (2 days/week) for 3 months [26]. The stimulation was applied over six brain regions, pinpointed with MRI in each patient, corresponding to specific cognitive functions: the left inferior frontal gyrus (known as Broca’s area) and the left superior temporal gyrus (known as Wernicke’s area) for language, the left and right DLPFC for judgment, executive functions and long-term memory, and the left and right parietal somatosensory association cortices for spatial/topographical orientation and “praxis.” The stimulation was combined with computerized cognitive training that included specific tasks on the same functions. They then used a variety of different indices, such as the well-known ADAS-Cog, the Clinical Global Impression of Change (CGIC),the MMSE and the Hamilton Depression Scale (HAL-D), examining the patients after 6 weeks and 4.5 months from the initiation of the stimulation sessions. ADAS-Cog and CGIC scores significantly improved at both assessment times, while most of the other indices also improved but without attaining statistical significance. Two years later, the same group conducted a double-blinded controlled study with 15 AD patients [14]. Seven of those received real HF-rTMS (10 Hz, 90–110% MT intensity depending on the region) over the aforementioned regions and cognitive training for 6 weeks (5/week) followed by two weekly sessions for 3 months, whereas eight received sham stimulation alternatively. After 6 weeks of treatment, the ADAS-Cog scores had significantly improved for the real treatment group when compared to sham, and after 4.5 months, they remained improved, while the scores deteriorated for the sham group. CGIC scores also demonstrated significant improvement. In both studies, patients were under treatment (mostly with cholinesterase inhibitors), something that suggests that rTMS can provide additional benefits to pharmacological treatments. Finally, the group published another study on the joint effects of rTMS–cognitive training [29]. They included 30 mild to moderate AD patients that underwent the same 6-week protocol as described before, with the patients receiving cognitive training while the respective area was being stimulated. Afterwards, tests designed to assess the respective cognitive functions were performed. The researchers reported that ADAS-Cog and MMSE scores significantly improved after the treatment when compared to baseline, while approximately 80% of the patients showed improvement with the stimulation. Five of the patients were also summoned for a second round of treatment approximately 10 months after the first, and the prolonged effect of the first treatment round was assessed. After the second round, the cognitive results were the same or even slightly better than the first, showing that patients did not deteriorate in that 10-month interval after the intervention. Of course, as mentioned before, due to the same group conducting these studies, the results need to be regarded with more caution.

Further enhancing the positive impact of combined rTMS and cognitive training, a poster by Brem et al. (2013) refers to patients with mild AD (number not specified) that received either HF-rTMS (20 Hz, 120% MT intensity) over the same six regions (three per session, randomly selected each time) and concomitant cognitive training for 6 weeks, or sham stimulation and training [27]. Within the first month, patients receiving real treatment showed significant ADAS-Cog improvement compared to sham, and non-significant improvement in MMSE and CGIC. The same group recently published the results of a trial involving 34 AD patients, randomized to receive real or sham HF-rTMS (10 Hz, 80% AMT intensity) over the aforementioned six regions, and real or sham computerized cognitive training [32]. The patients were primarily assessed with the ADAS-Cog scale before, 1 week after and 4–6 weeks after the intervention. Additionally, the Geriatric Depression Scale (GDS) and the Clinical Dementia Rating scale (CDR) were evaluated at baseline, and the Clinical Global Impression of Change (ADCS-CGIC) was administered after the stimulation as well. Overall, directly after the intervention, patients in the real/real group showed greater improvement in ADAS-Cog scores compared to real/sham and sham/sham groups, as neither sham groups had any significant improvement. The real/real group also continued to improve in the follow-up period, and no statistically significant differences in improvement were noted between real/real and real/sham, showing how “removing” the effect of cognitive training did not affect improvement. Upon the combination of sham groups versus the real/real group in the analyses, the improvement of ADAS-CGIC scores also became significant, further highlighting the importance of adding rTMS in order to enhance the training’s efficiency. Furthermore, the reported changes could not be attributed to a possible effect of the intervention to depression metrics.

Similarly, Lee et al. (2016) randomized 27 mild to moderate AD patients, 18 to real stimulation and 8 to sham (with one withdrawal) [28]. They kept the same protocols as the previously described studies with the six regions receiving HF-rTMS (10 Hz, 90–110% MT intensity) combined with cognitive training for 6 weeks (5/week), and clinical assessment with the same indices at baseline and at 6 weeks. The ADAS-Cog significantly improved in the real stimulation group, while MMSE and CGIC scores also improved. Additionally, the effects were stronger for the mild AD patients, particularly regarding language and memory. Nguyen et al. (2017) published the results of 10 patients receiving five sessions of HF-rTMS (10 Hz, 100% RMT intensity) over the described areas every week for 5 weeks [30]. Patients were assessed immediately after the protocol ended and after 6 months, by means of MMSE, ADAS-Cog and other scales pertaining to caretaker burden, apathy, locomotor activity and patient dependence. Setting a goal of ameliorating short-term memory, a function tied to the DLPFC, the researchers administered additional stimulation–training sessions over this area every day, either left or right. After the treatment, ADAS-Cog scores were significantly improved, but at 6 months, only the patients with the greatest improvement (>13% improved ADAS-Cog scores) had maintained improved scores. Apathy and dependence scores were found significantly improved throughout all the assessment timepoints. However, these results should be interpreted with caution, as no control group was available, and it is possible that the reported amelioration is the result of a placebo effect or a test–retest learning effect. 

Finally, Sabbagh et al. (2020) recently published the results of their phase III randomized, double-blinded, sham-controlled, clinical trial regarding this combination of rTMS and cognitive training [31]. They enrolled a fair number of mild to moderate AD patients and in the final analysis, 59 patients for real (with the same aforementioned parameters) and 59 for sham were included. Patients with better cognitive performance at baseline (ADAS-Cog<30) significantly improved after real stimulation compared to sham and compared to those with scores >30. Additionally, patients in the active group maintained their improvement at a 12-week follow-up assessment, while those having received sham returned to baseline scores. CGIC scores were also significantly different between real and sham, favoring real stimulation, at the 12-week follow-up point. It is interesting to note that, in the active group, only 16% showed a deterioration in CGIC scores, compared to 41.8% for sham, while only 11% with baseline ADAS-Cog<30 from the active group deteriorated, compared to 40% of the same subgroup in the sham branch.

In summary, it appears that this particular combination of HF-rTMS and cognitive training is an effective modality, as all available studies reported beneficial effects and positive results which also seem to persist over time. It is therefore very promising for the future; the areas/regions associated with the impaired language or cognitive functions of the patients could be targeted via rTMS while the patient is simultaneously receiving focused cognitive training. Additionally, it can complement medication, probably producing even better results; several of the studies compared the degree of improvement noted with their intervention to the degree of improvement noted with pharmaceutical agents, rTMS or cognitive training alone, as expressed with the same indices through studies, and showed that it was more effective in the combined condition. Nevertheless, more studies, preferably randomized, controlled and double-blinded, are needed, in order to elucidate the specific effects of rTMS and to accurately identify the most suitable stimulation parameters to secure optimal outcomes.

However, this particular combination protocol must be compared to simpler protocols in order to confirm its superiority, since studies only involving the DLPFC have also produced encouraging results. One study addressed this issue by comparing HF-rTMS (5 Hz, 100% MT intensity) over the left DLPFC to the six areas described above [40]. The researchers randomized 10 participants to DLPFC stimulation and 9 to the six areas of stimulation. They assessed the patients after the stimulation protocol and 4 months after its completion, using ADAS-Cog, MMSE, CGI and other scores pertaining to behavioral and depressive symptoms. In both groups, scores were significantly improved directly after the treatment and at follow-up, while no differences between the protocols were noted in any of the scores. Thus, the authors suggested that the beneficial effect stemming from the more complex protocol is mostly the result of the DLPFC stimulation, an area critical to network integration. However, this study did not involve the cognitive training usually combined with the stimulation of these six areas, and whose therapeutic effect should not be underestimated [17]. On the other hand, acknowledging the complexity of the NeuroAD™ protocol, and the evidence to suggest that only stimulation of the DLPFC suffices to produce beneficial results, Bagattini et al. (2020) wished to further study the combination of cognitive training with rTMS over the left DLPFC only [41]. They conducted a randomized, double-blind, sham-controlled study, allocating 27 patients with either amnesic mild cognitive impairment (MCI) or mild to moderate AD to receive cognitive training directly after real rTMS (20 Hz, 100% RMT intensity), and 23 patients to receive cognitive training after sham rTMS. The RehaCom software was used for the computerized cognitive training sessions, which focused on face–name associative memory. The stimulation was administered 5 days per week for 4 weeks and patients were evaluated at baseline, at 4 weeks and at 12 weeks, by means of MMSE, Geriatric Depressive Scale (GDS) and other tests for specific cognitive functions such as memory, language, attention, spatial reasoning and praxis.The cognitive training significantly ameliorated face–name associative memory, while real stimulation provided significant additional benefits to associative memory, with this improvement being greater for patients with milder disease and higher levels of education. The real group also displayed improved non-trained visuospatial reasoning than the sham group, and this improvement was maintained when assessed at 12 weeks. This study showcases how rTMS can be an important add-on to cognitive training, but since both groups received cognitive training, it fails to provide additional information on whether rTMS is beneficial as a standalone treatment, as the studies analyzed before reported. As such, more studies comparing the available rTMS methods, and their interaction with cognitive training, are warranted.

### 2.3. Other Areas/Protocols

Koch et al. (2018) enrolled subjects with prodromal AD in order to investigate the effect of stimulation over the precuneus, an area of the parietal lobe thought to be implicated in AD-related memory deficits in early disease stages, due to large neuronal network connectivity impairments [42]. In this double-blinded, randomized, sham-controlled study, seven patients received HF-rTMS (20 Hz, 100% RMT intensity) over the precuneus bilaterally for 10 daily sessions in the span of two weeks, and seven patients received sham stimulation. After a two-week period, the patients were crossed over to the other experiment branch. It is of note that this study also used biomarkers to confirm the diagnosis of prodromal AD, and additionally paired TMS with EEG to uncover the neurophysiological effects of their stimulation. Cognitive assessments were performed with the Alzheimer Disease Cooperative Study Preclinical Alzheimer Cognitive Composite, before and after every two-week protocol. Real stimulation significantly improved episodic memory, with no differences noted for other cognitive functions between real and sham. Neurophysiologically, rTMS enhanced functional connections between the precuneus and medial frontal areas. Increased activity in the precuneus is associated with memory retrieval, while decreased activity is shown during memory encoding. This “encoding/retrieval flip” has been shown to suffer in older individuals with amyloid accumulation [43]. As such, precuneus function enhancement via HF-rTMS expectedly led to memory improvement, as shown in this study.

Avirame et al. (2016) employed deep TMS (dTMS), a method that uses a particular type of coil to reach deep cortical regions, in an attempt to stimulate the prefrontal cortex (PFC) of AD patients [44]. They enrolled 11 patients with moderate to severe AD, who received 20 sessions of dTMS (10 Hz, 100–120% MT intensity) over the PFC bilaterally, assessed by means of Mindstreams (MS) and Addenbrooke Cognitive Examination (ACE) scores before and after the stimulation protocol. An improvement was reported for 60% and 77%of the patients in MS and ACE scores respectively, with this improvement approaching significance. Significance was reached when six patients with more severe disease were separately analyzed. Additionally, improvement in visuospatial abilities was significant, with attention and executive function approaching the threshold as well.

Anderkova et al. (2015) performed an interesting study on how brain atrophy impacts the effectiveness of rTMS [45]. They enrolled 20 patients with mild AD and performed three sessions of HF-rTMS (10 Hz, 90% RMT intensity) over the right inferior frontal gyrus (IFG), the right superior temporal gyrus (STG) and a sham stimulation in a randomized order, and with an interval of at least one day before switching to a different branch. The patients were assessed with the Trail Making Test (TMT), the Stroop Test (ST), the Complex Visual Scene Encoding Task (CVSET) and the MMSE before and after each treatment. Significant improvements on the word part of the ST were noted for both STG and IFG stimulations, with IFG also significantly improving TMT performance; this translates into better attention and psychomotor speed. Regarding atrophy, patients exhibited characteristic patterns of atrophy compared to controls, and a specific pattern of gray matter atrophy correlated with the diminished effectiveness of rTMS on word scores of the ST. This shows how several parameters may affect rTMS effectiveness, and as such studies like this help in stratifying patients more likely to be assisted by the intervention.

Zhao et al. (2017) randomized 30 mild to moderate AD patients (17 for real and 13 for sham stimulation), and applied HF-rTMS (20 Hz, intensity not specified) over three brain areas (parietal P3/P4 and posterior temporal T5/T6, third area not specified) in daily sessions for 6 weeks [46]. The patients were assessed before the protocol, immediately afterwards, and 6 weeks later by means of MoCA, ADAS-Cog, MMSE and World Health Organization University of California-Los Angeles, Auditory Verbal Learning Test (WHO-UCLA AVLT) scores. ADAS-Cog, MMSE and WHO-UCLA AVLT scores were significantly improved at 6 weeks after the intervention, while MoCA scores were significantly improved for the mild subgroup. Additionally, ADAS-Cog scores for moderate patients alone did not significantly improve compared to sham.

## 3. Mild Cognitive Impairment and Aging

Some studies have included subjects with mild cognitive impairment (MCI); in this condition, individuals do present memory impairment, either subjective or objective, but that is not enough to disturb their daily activities or to fulfill the diagnostic criteria for dementia. However, a fair percentage of MCI patients later end up developing dementia, primarily AD [47]. Consequently, studies on this patient subgroup are also of importance, since impairments are present, and this entity may represent an early stage of dementia as well. Two relevant studies have already been described in the previous section.

First, Cotelli et al. (2012) described the case of an 81-year-old man with amnesic MCI [48]. After two online rTMS sessions to pinpoint the location they would consistently stimulate, they found that only stimulation of the left inferior parietal cortex (IPL) improved accuracy in FNAT (Face–Name Association Test) scores. Subsequently, the patient received HF-rTMS (20 Hz, 100% MT intensity) stimulation over that area for 2 weeks (5/week). A significant improvement in FNAT scores was noted upon completion of the 2 weeks, so the patient exhibited better memory functions, and this change was also evident when the patient was assessed at 24 weeks follow-up. Eliasova et al. (2014) randomized 10 amnesic MCI/AD patients into one group receiving real HF-rTMS (10 Hz, 90% MT intensity) over the right IFG and one group receiving sham treatment [49]. The patients were then assessed with the TMT-A and -B (testing visuospatial processing speed and cognitive flexibility skills, respectively), the ST and the CVSET, before and after the stimulation. Significant effects were noted for both parts of TMT, showing improved attention and psychomotor speed. 

DrumondMarra et al. (2015) conducted another randomized, double-blinded, controlled study, by randomizing 34 elderly patients with MCI into either receiving ten sessions of active HF-rTMS (10 Hz, 110% MT intensity) over the left DLPFC (15 patients), or sham (19 patients) [50]. The patients were assessed at baseline, right after the intervention and after one month. Everyday memory improvement, measured by the Rivermead Behavioral Memory Test (RBMT), was noted for the active stimulation, which persisted after one month.

Padala et al. (2018) studied the effects of HF-rTMS (10 Hz, 120% MT) over the left DLPFC in nine MCI patients in order to assess its effectiveness on apathy, an important neurobehavioral aspect of several neurodegenerative conditions [51]. Patients were randomized to either real or sham stimulation (5 days per week for 2 weeks) and were then crossed over to the other branch after an interval of one month. Apathy, executive function and cognition were assessed at baseline, after the interventions, and after the interval. Significant improvement in all these domains was noted after real stimulation, suggesting that rTMS is an attractive option for apathy, a condition inherently difficult to handle pharmacologically [52].

In an attempt to elucidate the mechanisms of rTMS’s efficacy and its effects on neural network connectivity, Cui et al. (2019) enrolled 25 MCI patients (21 completed the protocol) in their double-blind, sham-controlled study [53]. They targeted the right DLPFC, as part of the so-called ‘default mode network’ (DMN), a constellation of functionally connected brain areas that seem to be silenced during attention-requiring tasks, and to represent the brain’s intrinsic organization [54]. In this study, the patients were randomized to either receive HF-rTMS (10 Hz, 90% RMT) or sham for two weeks (5 days/week), and to be assessed directly and two months after its completion via MMSE, ACE-III, GDS and other tests for particular cognitive domains. They reported that real stimulation improved immediate and delayed free recall, and this improvement persisted in the follow-up assessment. This study also included fMRI (functional magnetic resonance imaging) to assess activity within areas of the DMN, and reported that subjects with lower activity levels at baseline presented higher responsiveness to treatment. 

Finally, two studies enrolled aging individuals; one healthy and one presenting memory impairment. Normal aging entails a plethora of pathological alterations that also resemble AD, such as ER dysfunction [16], and so the effects of rTMS on aging individuals present a certain interest regarding dementia as well.

Kim et al. (2012) enrolled healthy aging individuals, as, per their rationale, the effect of rTMS had not been investigated in this population [55]. Subjects with concurrent pathologies are more frequently involved in the relevant literature, although normal aging is also associated with faultier selection processes and greater attention deficits upon exposure to task-irrelevant stimuli, alongside other impaired cognitive functions [56]. Thus, the researchers assigned eight individuals into a real stimulation group, receiving HF-rTMS (10 Hz) over the left DLPFC for 5 consecutive days, and eight into a sham group. They used the ST for inhibition control assessment, one day before and one day after the intervention. Those receiving real stimulation showed improvement in task performance, showing that rTMS can prove beneficial even in normal aging. Solé-Padullés et al. (2006) enrolled 40 participants over the age of 50, who complained of memory difficulties and had memory performance within the lower normal range (therefore not fulfilling any dementia criteria) [57]. They then randomized them to one group receiving real HF-rTMS over the right and left DLPFC (10 Hz, 80% MT intensity), and one sham group, and assessed them with FNAT, in an offline design. Only those in the real stimulation group showed significant improvement in associative memory. Further analysis with fMRI demonstrated the recruitment of supplementary regions in the right prefrontal and the posterior cortical areas of both hemispheres, implying that rTMS enhances the activation of these additional areas to facilitate memory functions.

Collectively, these studies show that rTMS can be proven beneficial even for healthy individuals or those with mild disturbances, further highlighting its effectiveness in ameliorating cognitive functions. We believe that longitudinal, sham-controlled studies with patients with MCI/memory complaints that follow an rTMS protocol could help determine whether this early intervention is capable of preventing or delaying full-scale dementia.

## 4. Frontotemporal Dementia

Frontotemporal dementia (FTD) is a frequent dementia type in individuals below the age of 65, and usually leads to death in less than 10 years. It is characterized by neurodegeneration in the frontal and/or temporal lobes, with a wide array of atrophy patterns, and symptom constellations that include personality alterations, behavioral disorders and language and executive function impairments [58,59]. The main recognized subtypes are the behavioral variant (bvFTD), featuring lack of inhibition, compulsive behavior, personality changes, and Primary Progressive Aphasia (PPA), a syndrome that mostly affects language skills. PPA has three recognized subtypes: the non-fluent/agrammatic variant (nfvPPA), the semantic variant-primary progressive aphasia (svPPA), and the logopenic variant (LPPA) [59,60]. Behavioral and psychological symptoms are usually treated with selective serotonin reuptake inhibitors (SSRIs) and atypical antipsychotics, but no treatment is available for the cognitive deficits [58]. As such, non-pharmacological options are also being explored for this dementia.

Finocchiaro et al. (2006) first reported the use of HF-rTMS (20 Hz, 90% MT intensity) on a 60-year-old right-handed PPA patient with bilateral frontotemporal atrophy, more pronounced on the left hemisphere [61]. They administered two sessions of real HF-rTMS over the left PFC, and one session of sham, assessing the patient with several memory and language tests before and after the sessions. Verb production was significantly enhanced after real stimulation. In a similar vein, Trebbastoni et al. (2013) published another case report on PPA, employing deep HF-rTMS (20 Hz, 100% RMT intensity) and sham over the left DLPFC of a right-handed 50-year-old patient with phonological errors, impaired word recall and sentence repetition, alongside perisylvian atrophy and hypoperfusion, all key features of LPPA [62]. He received two consecutive 5-day real rTMS sessions, and two of sham, and was evaluated before and after with a variety of tasks assessing frontal, language and visuospatial functions. A significant improvement was noted for the language domain after real stimulation. These two case reports suggest that in the setting of PPA, rTMS seems to selectively improve language function, which is the function most heavily impaired in PPA.

Antczak et al. (2018) conducted a pilot study on HF-rTMS for FTD, by enrolling nine patients with bvFTD, one with nfvPPA and one with progressive nfvPPA [58]. The patients received 10 sessions of HF-rTMS (10 Hz, 90% RMT intensity) over the bilateral DLPFC in two weeks, and were cognitively and behaviorally assessed before and after the treatment by means of CGIS, the 21-item HDRS, Geriatric Depression Scale (GDS), Frontal Assessment Battery (FAB) and MoCA. After the intervention, total MoCA score, visuospatial performance and Stroop test subscores (reading time and error number) were improved. Additionally, two out of the three patients with mild depression were shown to return to normal, while a patient with severe depression was afterwards classified as mild.

Here, we deem it useful to mention that a larger, randomized, sham-controlled study on the use of a different form of brain stimulation, the transcranial direct current stimulation, has been recently published [63]. Fifty-five patients and 15 presymptomatic individuals were enrolled, and the left prefrontal cortex was targeted. Improvement in clinical scores and behavioral symptoms was noted after the real stimulation in both groups, alongside an increase in intracortical connectivity. This study exceeds the purposes of the current review, but enhances the notion that non-invasive brain stimulation can be a useful modality for FTD.

This preliminary evidence suggests that rTMS may eventually hold an important position in treating FTD. Understandably, a single study that did not include controls and two case reports are less than enough to reach safe conclusions; this field warrants more research, since this disease affects relatively young individuals who are considerably impaired in their daily functions, with no effective pharmacological treatment available.

## 5. Vascular Dementia

The second commonest dementia in older ages is vascular dementia (VD), which overlaps with AD in many patients. It stems from progressively acquired ischemic, hypoxic or hemorrhagic brain lesions as a result of cardiovascular and cerebrovascular disorders [64]. VD and AD share several risk factors, such as hypertension and diabetes, but can be clinically differentiated by the fact that in VD, executive dysfunction is usually the first to appear, and cognitive performance seems to fluctuate and worsen abruptly, instead of progressively declining, such as in AD. Mood and personality changes are also more severe in VD. It is of note that cholinergic deficits are noted in VD as well, and this possibly explains why cholinesterase inhibitors are also therapeutically used in this disease [64,65].

Despite it being the second commonest form of dementia and havinga similar pathophysiology to stroke, which has extensive rTMS literature [66], studies on the role of rTMS in VD are few.

Two animal models of VD showed that LF-rTMS (0.5 Hz) and HF-rTMS (5 Hz) significantly improved learning and memory, increased the density of cholinergic neurons and BDNF (brain-derived neurotrophic factor) in hippocampal CA1 area [64,67]. Regarding humans, only two cases have been published so far [65]. Two female patients with VD underwent 40 sessions of a commercially available protocol developed in Mexico, which was otherwise not specified and the stimulation parameters could not be found. The patients were assessed at baseline and two months later. The first patient showed a 7-point improvement in the MMSE and, reportedly, no language difficulty and better social interactions. The second patient showed a 10-point improvement in the MMSE score, with better social interactions and daily activity function.

Two studies involved individuals with known cerebrovascular disease that did not otherwise fulfill dementia criteria. In the earlier study [68], seven such patients with mild executive dysfunction were randomized and then crossed-over, to receive either HF-rTMS (10 Hz, 100% MT intensity) over the left DLPFC or the left motor cortex as a control, undergoing one session of each with a 3-day interval between sessions. They were assessed with a variety of neuropsychological tests, such as the TMT and the Stroop test, focused on psychomotor speed, memory and executive functions. The only significant improvement upon stimulation of the DLPFC was reported for the Stroop test, indicating amelioration in processing speed and attention. However, this study included a small number of patients, and a test–retest effect cannot be excluded either. Sedlackova et al. (2011) enrolled seven subjects with MCI of the vascular type without dementia, and tested HF-rTMS (10 Hz, 100% RMT intensity) and LF-rTMS (1 Hz, 100% RMT intensity) over the left DLPFC, and over the motor cortex as a control, in a crossover design [69]. Numerous short neuropsychological tests, such as the TMT, were then administered. No results in cognitive performance were noted for either intervention over the DLPFC.

As such, there is a great paucity of studies on VD, which we hope will be addressed in the near future by more studies providing knowledge currently lacking.

## 6. Lewy Body Dementia

Lewy body dementia (LBD) is the second commonest neurodegenerative dementia, and includes dementia with Lewy bodies (DLB) and Parkinson’s disease (PD) dementia (PDD) [70]. As evident from its name, the disease is pathologically characterized by Lewy body protein aggregations, and its symptoms, besides cognitive impairment, include Parkinsonism, serious behavioral and psychological disorders, vivid and recurrent hallucinations and severe sensitivity to antipsychotics [71]. No disease-modifying treatment is available for these diseases either, and limitations regarding the treatment of behavioral/psychological symptoms have directed scientific interest towards non-invasive methods [70].

RTMS has been extensively studied in the context of PD, and a recent meta-analysis on the effects of rTMS on the cognitive performance of PD patients reported that HF-rTMS over the DLPFC may indeed be beneficial [72]. Due to the similarities between LBD and PD, and the existing literature and evidence on rTMS’s efficacy on psychiatric disorders [73], it has long been hypothesized that rTMS could also be a therapeutic option for LBD [71]. However, only one study has involved rTMS in LBD, focusing on depression. In that study, rTMS was evaluated in six LBD patients with drug-resistant depression. The protocol involved daily sessions of LF-rTMS (1 Hz, 110% MT intensity) over the right DLPFC and HF-rTMS (10 Hz, 100% MT intensity) for the left DLPFC for ten days. Patients were assessed with HAL-D before and after the intervention, which was found to significantly improve depressive symptoms [74]. 

Finally, besides those analyzed in the aforementioned meta-analysis, another recent study explored rTMS in PDD [75]. The researchers randomized 33 PDD patients to receive either HF-rTMS (20 Hz, 90% RMT) (18 patients) over the hand area of both primary motor cortices for two weeks (5 days/week), or sham (15 patients). They further received monthly boosting sessions for 3 months. The patients were assessed with the MoCA, MMSE, CDR and Memory and Executive Screening (MES) and Instrumental Activity of Daily Living (IADL) scales. The rationale of the researchers was that improvement in the ability to move about the environment more freely would aid in improving cognition, and that the primary motor cortex is itself involved in some cognitive tasks, such as movement imagery, attention and language [76]. Only a small positive effect on MMSE, MoCA and IADL scores alongside an improvement in motor function was noted. Additionally, this improvement in cognition was not detected in the follow-up sessions, and improvements in MoCA and CDR scales significantly correlated with improvements in the motor assessment. As such, it is possible that the recorded positive effects reflect an influence of the motor cortex on cognitive processes, albeit a small one.

## 7. Ongoing Trials

Searching the clinicaltrials.gov website (last accessed on 7 June 2021) with the keywords “dementia” and “rTMS”, 36 results come up. Of these, one employed transcranial direct stimulation and was thus not further assessed, and from the remaining 35, after removing those with published results and those that were irrelevant, 25 remained and are presented in Table 3.

## 8. Discussion

RTMS represents a promising modality in treating a plethora of neurological and psychiatric disorders. Per the newest guidelines, it has received B level recommendation for its use in neurodegenerative disorders, namely PD and multiple sclerosis [73]. This is very encouraging, given that AD and dementias share several common elements with these disorders, such as their pathogenetic mechanisms [77,78]. Additionally, these diseases are known for their cognitive sequelae [79,80], and rTMS has been explored as a treatment modality for cognitive decline in this context as well [5,72]. As such, rTMS’s efficacy in this setting raises hopes for its application in dementia as well.

In the available literature, the vast majority of rTMS and dementia studies focus on AD. This is reasonable when one considers that is the most frequent dementia type, but the paucity of studies on other dementias highlights the need for additional research regarding these diseases, as many individuals are also heavily affected by them.

Regarding AD in particular, most studies have shown that HF-rTMS is beneficial, as it improves cognitive performance, measured by a variety of scores, and this effect is not only limited to specific domains, but overall daily functional capacity and quality of life. The DLPFC is the main area of interest in AD-and-rTMS literature, as many studies have either focused on it solely, or have included it in multiple-area protocols. However, the superiority of a more complex protocol over one only involving the DLPFC has yet to be proven. A commercially available system that includes rTMS over six areas of interest combined with respective cognitive training has been explored in several studies, with encouraging results [14,26,27,28,29,30,31,32], but only one publication compared rTMS over the DLPFC to rTMS over these six regions, and it did not show any difference [40]. This study did not include cognitive training, so whether the benefits of this system stem from the cognitive training only and not rTMS should be further explored.

The right DLPFC represents another “mystery.” It most likely negatively impacts cognition and memory, as its inhibition via LF-rTMS led to episodic memory improvement in both healthy and demented individuals [25,37]. It is likely that, in the protocols with both DLPFCs stimulated, the reported positive effects were the result of the left DLPFC being enhanced, which holds the greatest significance regarding language and memory [81], and this enhancement overcame the counteraction of the right DLPFC. However, the study of Cui et al. (2019) [53] reported that HF-rTMS over the right DLPFC led to improved immediate and delayed free recall, something that contradicts the aforementioned findings. Naturally, methodological differences, namely in the protocols and the tests administered, existed between the studies, and there are not many studies to pool together and draw an accurate conclusion.

A number of studies have assessed the combined efficacy of rTMS and cognitive training [14,26,27,28,29,30,31,32], but few attempted to compare the two and examine their interaction. Brem et al. (2020), showed that rTMS was crucial for the effects of cognitive training to become significant [32], supporting the notion that rTMS is the main player in improving cognitive performance and that cognitive training works as an add-on to its effects. On the contrary, Bagattini et al. (2020) showed that rTMS over the left DLPFC served as an add-on to cognitive training instead, improving associative memory and further providing a “generalization” effect, where improvement was noted in domains that had not been cognitively trained, regardless of real or sham rTMS allocation [41]. However, this should be interpreted with caution, since this study did not contain a group that received sham cognitive training and sham rTMS, and a learning effect cannot be excluded. Additionally, no studies directly comparing rTMS as a standalone treatment and pharmacological treatments have been conducted. In the majority of studies, the patients were receiving some sort of standard-of-care treatment for AD, so whether rTMS can be considered a monotherapy or an add-on to other treatments still remains a matter of debate, and studies for the immediate comparison of cognitive training, rTMS, medication and their interaction are required.

Another issue that frequently came up is the greater effectiveness that rTMS seems to have when applied at earlier disease stages. Several studies showed that patients in earlier stages (mild disease) had better responsiveness after treatment [22,23,28,41,46]. This finding is reciprocated by Sabbagh et al. (2020), who showed greater improvement for patients with better baseline scores [31]. Additionally, Nguyen et al. (2017) reported that only patients with the highest baseline scores maintained the improvement induced by rTMS in their follow-up assessment [30]. This phenomenon could also reflect the amount of brain atrophy present, since this worsens as the disease progresses, and Anderkova et al. (2015) showed that gray matter atrophy negatively impacted responsiveness to rTMS [45]. As another metric of disease progression, the employment of additional brain areas for specific tasks, can be detected via fMRI, and represents a compensatory mechanism [25,36]. In this regard, Cui et al. (2019) reported that MCI patients with lower baseline DMN activity benefited more from the intervention [53]. Only one study showed that the subgroup with the more severe disease course benefited more from the intervention [44]. However, this study employed deep TMS, which stimulates more deeply but in a less focused manner. As such, this method may fit better for individuals in more advanced stages, where the brain networks are more diffusely damaged. In any case, the available literature seems to agree that patients gain more out of the procedure when this is applied at earlier stages and when cognitive functions are better preserved. This phenomenon is congruent with the fact that rTMS has also proven beneficial for patients with MCI or generic memory complaints without a diagnosis of dementia, as we analyzed in the section above, and is further corroborated by the plethora of ongoing trials involving MCI individuals. However, the recruitment of patients with severe forms of dementia is more challenging than enrolling those at prodromal and early stages, so there is paucity of studies comparing an adequate number of severe and mild AD patients. In any case, the available results highlight the need for a timely and early intervention, so that the cognitive level may be preserved and even ameliorated. The exact protocols and the intervals for maintenance sessions still need to be determined, but rTMS in AD seems to be a very promising treatment option for the future. Additionally, rTMS in MCI is a very attractive research field, as the true potential of rTMS in delaying dementia progression or even ultimately preventing it can be revealed.

In a similar vein, the long-term efficacy of rTMS is another issue that needs to be discussed. Not all of the studies assessed the patients after the stimulation period ended, and those that did set heterogeneous timepoints, spanning from one or two months [20,21], to 3–4 months [22,26,31] to even 6 months [30]. The most ambitious study [23] involved a more longitudinal follow-up, ranging from 10 to 19 months. However, within this timeframe, many patients were lost to follow-up or ceased their sessions. This represents an issue in assessing the long-term efficacy of rTMS in dementia, since the patients are inherently hard to “maintain,” given their old age and burden of disease. Additionally, providing follow-up, boosting sessions also represents a challenge since patients need to be brought to the facility with the machinery, which is not always an easy task for the caregivers and the patients. However, the fact remains that even in these relatively short follow-up periods, some benefits from the intervention were maintained [14,22,26,29,31,32]. Two studies [30,31] reported maintenance, or maintenance at higher levels, of the beneficial effects for those with better baseline scores, further corroborating that rTMS is more efficacious in earlier disease stages. One study [29] re-summoned patients 10 months after their initial protocol. Those that participated in this second round were found to have the same or even better results than the first round. This shows how these patients, albeit few, had not deteriorated within this period, and that after an initial intensive protocol, boosting sessions can be set for a later point in time, possibly assisting in adherence to treatment.

The search for studies on rTMS and the remaining dementias has yielded very few results. For instance, it was very surprising to see that only two cases of rTMS having been applied in VD are available. rTMS is a very safe technique, with very few and minor side-effects, which are mostly self-resolved [4]. Bearing that in mind, the lack of data is rather intriguing. Additionally, in almost all of the aforementioned studies, no side-effect was severe enough to lead to the discontinuation of the protocol. As such, one can only stress how important more studies on these common dementia types and this modality are needed.

It is understandable that large-scale studies with considerable patient cohorts are not easily conducted. A significant limitation would be the lack of the proper equipment by the involved institutions. As such, a multicenter study design is recommended, for larger samples to be gathered and for more accurate conclusions to be drawn. Naturally, randomized, sham-controlled studies must be preferred, and it is imperative that clinical trials in the future are of high quality, in order to provide solid evidence for the efficacy of rTMS. Finally, as has been shown, several published trials in various disorders are of suboptimal quality [82,83], a fact that limits the applicability of their results. Indeed, several of the aforementioned studies had several vital parts of their methodology inadequately reported. Therefore, we propose adherence to the CONSORT statement, as a means ofensuring optimal reporting quality and minimizing bias.

Summing up, in light of the available information, it appears that rTMS holds promise in the amelioration of dementia symptomatology, especially in AD. However, the particulars of the “best” protocol have yet to be defined and high quality clinical trials are urgently needed to provide solid evidence inthis direction. Furthermore, research is still in embryonic stages regarding disorders such as LBD, FTD and VD; this is particularly disappointing considering there is a wealth of literature regarding the application of rTMS in the context of similar disorders such as PD and ischemic stroke [73]. Therefore, we hope that future research endeavors will be turned in this direction, which could help improve the lives of millions of patients suffering from dementia via a safe and effective non-pharmacological intervention.

## Figures and Tables

**Table 1:** rTMS over the DLPFC * in Alzheimer’s disease.

Reference	Protocol	Results
[18]	HF ** (20 Hz), bilaterally	Improved action naming
[19]	HF (20 Hz), bilaterally	Improved action naming in milder cognitive decline Improved general naming in moderate to severe cognitive decline
[20]	HF (20 Hz), left	Improved auditory sentence comprehension, persisted for 2 months
[21]	HF (10 Hz), left	Improved neuropsychological test scores and daily functioning
[22]	HF (20 Hz)/LF *^3^ (1 Hz), bilaterally	Improved cognitive function and mood in the HF group, persisted for 3 months
[23]	HF (20 Hz), bilaterally	Improved cognitive function and behaviorImproved word-image association
[24]	HF (20 Hz), left	Improved cognitive function and behavior
[25]	LF (1 Hz), right	Improved episodic memory (non-verbal recognition)

* Dorsolateral prefrontal cortex. ** High-frequency rTMS. *^3^ Low-frequency rTMS.

**Table 2:** rTMS over 6 areas of interest * combined with respective cognitive training in AD.

Reference	Protocol	Results
[26]	HF (10 Hz)	Improved cognitive function, persisted in the maintenance period
[14]	HF (10 Hz)	Improved cognitive function, persisted in the maintenance period
[27]	HF (20 Hz)	Improved cognitive function
[28]	HF (10 Hz)	Improved cognitive function, stronger results for milder cognitive decline
[29]	HF (10 Hz)	Improved cognitive function, persisted in the maintenance period
[30]	HF (10 Hz)	Improved cognitive function, persisted at 6 months for those with better baseline scores
[31]	HF (10 Hz)	Improved cognitive function that persisted, specifically for those with better baseline scores
[32]	HF (10 Hz)	Improved cognitive function, persisted in follow-up, no differences between groups receiving real or sham cognitive training

* Left inferior frontal gyrus, left superior temporal gyrus, left and right dorsolateral prefrontal cortex, left and right parietal somatosensory association cortices.

**Table 3:** Ongoing studies on the use of repetitive transcranial magnetic stimulation in dementia.

NCT Number	Dementia Type	Details
NCT02621424	MCI */AD **	Last update: May 2021, active—not recruiting Randomized, crossover, sham-controlled Target: DLPFC *^5^ Outcome: Cognitive score improvement and CSF *^6^ BDNF *^7^ levels
NCT01894620	AD	Last update: February 2021, completed, preliminary results listed Randomized, crossover, sham-controlled Outcome: Cognitive score and sleep improvement
NCT02537496	AD	Last update: February 2019, completed, no results listed Randomized, sham-controlled Target: Left DLPFC Outcome: Executive function/working memory improvement
NCT04562506	AD	Last update: September 2020, completed Randomized, sham-controlled, double-blinded Target: Bilateral DLPFC Outcome:Cognitivefunctions
NCT03665831	MCT/AD with comorbid MDD *^3^	Last update: October 2019, recruiting Open-label trial Target: Left DLPFC Outcome: Emotional/cognitive symptoms
NCT02908815	AD	Last update: February 2021, recruiting Randomized, sham-controlled Target: DLPFC Outcome: Cognitive functions
NCT01885806	AD-related apathy	Last update: June 2013, unknown Randomized, sham-controlled Target: Left DLPFC Outcome: Apathy symptoms
NCT04754152	MCI/AD	Last update: May 2021, recruiting Randomized, sham-controlled Outcome: Cognitive functions
NCT04012346	MCI/AD	Last update: July 2019, unknown Randomized, double-blinded, sham-controlled Outcome: Cognitive functions
NCT04042532	Early onset AD	Last update: April 2021, enrolling by invitation Randomized, double-blinded, sham-controlled Target: Left DLPFC Outcome: Cognitive functions
NCT04555941	MCI/AD	Last update: October 2020, recruiting Randomized, triple-blinded, sham-controlled Outcome: Cognitive functions
NCT01481961	Early AD	Last update: March 2015, completed Open-label trial Target: Left DLPFC Outcome: Cognitive functions
NCT03612622	MCI/Early AD	Last update: February 2021, completed Randomized, triple-blinded, sham-controlled Outcome: Associative memory/cognitive and psychological symptoms
NCT04440891	AD	Last update: April 2021, recruiting Randomized, double-blinded, sham-controlled Outcome: Cognitive functions
NCT03270137	AD	Last update: September2017, unknown Randomized, single-blinded Target: Left DLPFC/six region protocol Outcome: Cognitive functions
NCT04263194	Mild AD	Last update: August 2020, recruiting Randomized, triple-blinded, sham-controlled Target: DMN *^8^ Outcome: Cognitive symptoms
NCT03778151	Mild AD	Last update: February 2021, completed Randomized, double-blinded, sham-controlled Target: DMN Outcome: Cognitive symptoms
NCT04294888	Prodromal and Preclinical AD	Last update: March 2020, recruiting Randomized, cross-over, single-blinded, sham-controlled Target: DMN Outcome: Associative memory/functional connectivity
NCT04045990	Amnestic MCI/Logopenic PPA *^4^	Last update: November 2020, recruiting Cross-over, single-blinded, sham-controlled Target: DMN Outcome: Language/memory
NCT03406429	Agrammatic Non-Fluent PPA/Logopenic PPA	Last update: March 2021, recruiting Open-label, cross-over, sham-controlled Target: Left DLPFC Outcome: Language/functional connectivity and cortical thickness
NCT04188067	PPA	Last update: February 2021, recruiting Open-label, cross-over, sham-controlled Target: Left DLPFC Outcome: Language/functional connectivity
NCT04193267	Logopenic PPA	Last update: March 2021, recruiting Open-label trial Target: Left superior temporal gyrus Outcome: Language
NCT04431401	PPA	Last update: June 2020, not yet recruiting Randomized, triple-blinded, sham-controlled Outcome: Language/functional connectivity
NCT03153540	Agrammatic Non-Fluent PPA	Last update: January 2021, recruiting Randomized, cross-over, quadruple-blinded, sham-controlled Target: Dominant inferior frontal gyrus Outcome: Safety, tolerability/language/brain function
NCT03448133	PPA	Last update: June 2020, withdrawn

* Mild Cognitive Impairment. ** Alzheimer’s Disease. *^3^ Major Depressive Disorder *^4^ Primary Progressive Aphasia. *^5^ Dorsolateral Prefrontal Cortex. *^6^ Cerebrospinal Fluid. *^7^ Brain-Derived Neurotrophic Factor. *^8^ Default Mode Network.

## References

[1] Takizawa C., Thompson P.L., van Walsem A., Faure C., Maier W.C. (2015). Epidemiological and Economic Burden of Alzheimer’s Disease: A Systematic Literature Review of Data across Europe and the United States of America. J. Alzheimer’s Dis..

[2] Hsu W.-Y., Ku Y., Zanto T.P., Gazzaley A. (2015). Effects of Noninvasive Brain Stimulation on Cognitive Function in Healthy Aging and Alzheimer’s Disease: A Systematic Review and Meta-Analysis. Neurobiol. Aging.

[3] Barker A.T., Jalinous R., Freeston I.L. (1985). Non-Invasive Magnetic Stimulation of Human Motor Cortex. Lancet.

[4] Rossi S., Hallett M., Rossini P.M., Pascual-Leone A. (2009). Safety of TMS Consensus Group Safety, Ethical Considerations, and Application Guidelines for the Use of Transcranial Magnetic Stimulation in Clinical Practice and Research. Clin.Neurophysiol..

[5] Aloizou A., Pateraki G., Anargyros K., Siokas V., Bakirtzis C., Liampas I., Nousia A., Nasios G., Sgantzos M., Peristeri E. (2021). Transcranial Magnetic Stimulation (TMS) and Repetitive TMS in Multiple Sclerosis. Rev. Neurosci..

[6] Hallett M. (2007). Transcranial Magnetic Stimulation: A Primer. Neuron.

[7] Hoogendam J.M., Ramakers G.M.J., Di Lazzaro V. (2010). Physiology of Repetitive Transcranial Magnetic Stimulation of the Human Brain. Brain Stimul..

[8] Nasios G., Messinis L., Dardiotis E., Papathanasopoulos P. (2018). Repetitive Transcranial Magnetic Stimulation, Cognition, and Multiple Sclerosis: An Overview. Behav. Neurol..

[9] Klomjai W., Katz R., Lackmy-Vallée A. (2015). Basic Principles of Transcranial Magnetic Stimulation (TMS) and Repetitive TMS (RTMS). Ann. Phys. Rehabil. Med..

[10] Siokas V., Aslanidou P., Aloizou A.-M., Peristeri E., Stamati P., Liampas I., Arseniou S., Drakoulis N., Aschner M., Tsatsakis A. (2020). Does the CD33 Rs3865444 Polymorphism Confer Susceptibility to Alzheimer’s Disease?. J. Mol. Neurosci..

[11] Stamati P., Siokas V., Aloizou A.-M., Karampinis E., Arseniou S., Rakitskii V.N., Tsatsakis A., Spandidos D.A., Gozes I., Mitsias P.D. (2019). Does SCFD1 Rs10139154 Polymorphism Decrease Alzheimer’s Disease Risk?. J. Mol. Neurosci..

[12] Aloizou A.-M., Siokas V., Vogiatzi C., Peristeri E., Docea A.O., Petrakis D., Provatas A., Folia V., Chalkia C., Vinceti M. (2020). Pesticides, Cognitive Functions and Dementia: A Review. Toxicol. Lett..

[13] Sami N., Rahman S., Kumar V., Zaidi S., Islam A., Ali S., Ahmad F., Hassan M.I. (2017). Protein Aggregation, Misfolding and Consequential Human Neurodegenerative Diseases. Int. J. Neurosci..

[14] Rabey J.M., Dobronevsky E., Aichenbaum S., Gonen O., Marton R.G., Khaigrekht M. (2013). Repetitive Transcranial Magnetic Stimulation Combined with Cognitive Training Is a Safe and Effective Modality for the Treatment of Alzheimer’s Disease: A Randomized, Double-Blind Study. J. Neural Transm..

[15] Jan A.T., Azam M., Rahman S., Almigeiti A.M.S., Choi D.H., Lee E.J., Haq Q.M.R., Choi I. (2017). Perspective Insights into Disease Progression, Diagnostics, and Therapeutic Approaches in Alzheimer’s Disease: A Judicious Update. Front. Aging Neurosci..

[16] Rahman S., Archana A., Jan A.T., Minakshi R. (2018). Dissecting Endoplasmic Reticulum Unfolded Protein Response (UPRER) in Managing Clandestine Modus Operandi of Alzheimer’s Disease. Front. Aging Neurosci..

[17] Nousia A., Siokas V., Aretouli E., Messinis L., Aloizou A.-M., Martzoukou M., Karala M., Koumpoulis C., Nasios G., Dardiotis E. Beneficial Effect of Multidomain Cognitive Training on the Neuropsychological Performance of Patients with Early-Stage Alzheimer’s Disease. https://www.hindawi.com/journals/np/2018/2845176/.

[18] Cotelli M., Manenti R., Cappa S.F., Geroldi C., Zanetti O., Rossini P.M., Miniussi C. (2006). Effect of Transcranial Magnetic Stimulation on Action Naming in Patients with Alzheimer Disease. Arch. Neurol..

[19] Cotelli M., Manenti R., Cappa S.F., Zanetti O., Miniussi C. (2008). Transcranial Magnetic Stimulation Improves Naming in Alzheimer Disease Patients at Different Stages of Cognitive Decline. Eur. J. Neurol..

[20] Cotelli M., Calabria M., Manenti R., Rosini S., Zanetti O., Cappa S.F., Miniussi C. (2011). Improved Language Performance in Alzheimer Disease Following Brain Stimulation. J. Neurol. Neurosurg. Psychiatry.

[21] Haffen E., Chopard G., Pretalli J.-B., Magnin E., Nicolier M., Monnin J., Galmiche J., Rumbach L., Pazart L., Sechter D. (2012). A Case Report of Daily Left Prefrontal Repetitive Transcranial Magnetic Stimulation (RTMS) as an Adjunctive Treatment for Alzheimer Disease. Brain Stimul..

[22] Ahmed M.A., Darwish E.S., Khedr E.M., El Serogy Y.M., Ali A.M. (2012). Effects of Low versus High Frequencies of Repetitive Transcranial Magnetic Stimulation on Cognitive Function and Cortical Excitability in Alzheimer’s Dementia. J. Neurol..

[23] Rutherford G., Lithgow B., Moussavi Z. (2015). Short and Long-Term Effects of RTMS Treatment on Alzheimer’s Disease at Different Stages: A Pilot Study. J. Exp. Neurosci..

[24] Wu Y., Xu W., Liu X., Xu Q., Tang L., Wu S. (2015). Adjunctive Treatment with High Frequency Repetitive Transcranial Magnetic Stimulation for the Behavioral and Psychological Symptoms of Patients with Alzheimer’s Disease: A Randomized, Double-Blind, Sham-Controlled Study. Shanghai Arch. Psychiatry.

[25] Turriziani P., Smirni D., Mangano G.R., Zappalà G., Giustiniani A., Cipolotti L., Oliveri M. (2019). Low-Frequency Repetitive Transcranial Magnetic Stimulation of the Right Dorsolateral Prefrontal Cortex Enhances Recognition Memory in Alzheimer’s Disease. J. Alzheimer’s Dis..

[26] Bentwich J., Dobronevsky E., Aichenbaum S., Shorer R., Peretz R., Khaigrekht M., Marton R.G., Rabey J.M. (2011). Beneficial Effect of Repetitive Transcranial Magnetic Stimulation Combined with Cognitive Training for the Treatment of Alzheimer’s Disease: A Proof of Concept Study. J. Neural Transm..

[27] Brem A.-K., Schilberg L., Freitas C., Atkinson N., Seligson E., Pascual-Leone A. (2013). Effects of Cognitive Training and RTMS in Alzheimer’s Disease. Alzheimer’s Dement..

[28] Lee J., Choi B.H., Oh E., Sohn E.H., Lee A.Y. (2016). Treatment of Alzheimer’s Disease with Repetitive Transcranial Magnetic Stimulation Combined with Cognitive Training: A Prospective, Randomized, Double-Blind, Placebo-Controlled Study. J. Clin. Neurol..

[29] Rabey J.M., Dobronevsky E. (2016). Repetitive Transcranial Magnetic Stimulation (RTMS) Combined with Cognitive Training Is a Safe and Effective Modality for the Treatment of Alzheimer’s Disease: Clinical Experience. J. Neural Transm..

[30] Nguyen J.-P., Suarez A., Kemoun G., Meignier M., Le Saout E., Damier P., Nizard J., Lefaucheur J.-P. (2017). Repetitive Transcranial Magnetic Stimulation Combined with Cognitive Training for the Treatment of Alzheimer’s Disease. Neurophysiol. Clin..

[31] Sabbagh M., Sadowsky C., Tousi B., Agronin M.E., Alva G., Armon C., Bernick C., Keegan A.P., Karantzoulis S., Baror E. (2020). Effects of a Combined Transcranial Magnetic Stimulation (TMS) and Cognitive Training Intervention in Patients with Alzheimer’s Disease. Alzheimers Dement..

[32] Brem A.-K., Di Iorio R., Fried P.J., Oliveira-Maia A.J., Marra C., Profice P., Quaranta D., Schilberg L., Atkinson N.J., Seligson E.E. (2020). Corticomotor Plasticity Predicts Clinical Efficacy of Combined Neuromodulation and Cognitive Training in Alzheimer’s Disease. Front. Aging Neurosci..

[33] Trojano L., Conson M., Maffei R., Grossi D. (2006). Categorical and Coordinate Spatial Processing in the Imagery Domain Investigated by RTMS. Neuropsychologia.

[34] Bai F., Zhang Z., Watson D.R., Yu H., Shi Y., Yuan Y., Zang Y., Zhu C., Qian Y. (2009). Abnormal Functional Connectivity of Hippocampus during Episodic Memory Retrieval Processing Network in Amnestic Mild Cognitive Impairment. Biol. Psychiatry.

[35] Smith G.E., Pankratz V.S., Negash S., Machulda M.M., Petersen R.C., Boeve B.F., Knopman D.S., Lucas J.A., Ferman T.J., Graff-Radford N. (2007). A Plateau in Pre-Alzheimer Memory Decline: Evidence for Compensatory Mechanisms?. Neurology.

[36] Grady C.L., McIntosh A.R., Beig S., Craik F.I. (2001). An Examination of the Effects of Stimulus Type, Encoding Task, and Functional Connectivity on the Role of Right Prefrontal Cortex in Recognition Memory. Neuroimage.

[37] Turriziani P., Smirni D., Zappalà G., Mangano G.R., Oliveri M., Cipolotti L. (2012). Enhancing Memory Performance with RTMS in Healthy Subjects and Individuals with Mild Cognitive Impairment: The Role of the Right Dorsolateral Prefrontal Cortex. Front. Hum. Neurosci..

[38] Strafella A.P., Paus T., Barrett J., Dagher A. (2001). Repetitive Transcranial Magnetic Stimulation of the Human Prefrontal Cortex Induces Dopamine Release in the Caudate Nucleus. J. Neurosci..

[39] Miniussi C., Cappa S.F., Sandrini M., Rossini P.M., Rossi S. (2003). The Causal Role of the Prefrontal Cortex in Episodic Memory as Demonstrated with RTMS. Suppl. Clin. Neurophysiol..

[40] Alcalá-Lozano R., Morelos-Santana E., Cortés-Sotres J.F., Garza-Villarreal E.A., Sosa-Ortiz A.L., González-Olvera J.J. (2018). Similar Clinical Improvement and Maintenance after RTMS at 5 Hz Using a Simple vs. Complex Protocol in Alzheimer’s Disease. Brain Stimul..

[41] Bagattini C., Zanni M., Barocco F., Caffarra P., Brignani D., Miniussi C., Defanti C.A. (2020). Enhancing Cognitive Training Effects in Alzheimer’s Disease: RTMS as an Add-on Treatment. Brain Stimul..

[42] Koch G., Bonnì S., Pellicciari M.C., Casula E.P., Mancini M., Esposito R., Ponzo V., Picazio S., Di Lorenzo F., Serra L. (2018). Transcranial Magnetic Stimulation of the Precuneus Enhances Memory and Neural Activity in Prodromal Alzheimer’s Disease. Neuroimage.

[43] Vannini P., Hedden T., Becker J.A., Sullivan C., Putcha D., Rentz D., Johnson K.A., Sperling R.A. (2012). Age and Amyloid-Related Alterations in Default Network Habituation to Stimulus Repetition. Neurobiol. Aging.

[44] Avirame K., Stehberg J., Todder D. (2016). Benefits of Deep Transcranial Magnetic Stimulation in Alzheimer Disease: Case Series. J. ECT.

[45] Anderkova L., Eliasova I., Marecek R., Janousova E., Rektorova I. (2015). Distinct Pattern of Gray Matter Atrophy in Mild Alzheimer’s Disease Impacts on Cognitive Outcomes of Noninvasive Brain Stimulation. J. Alzheimer’s Dis..

[46] Zhao J., Li Z., Cong Y., Zhang J., Tan M., Zhang H., Geng N., Li M., Yu W., Shan P. (2017). Repetitive Transcranial Magnetic Stimulation Improves Cognitive Function of Alzheimer’s Disease Patients. Oncotarget.

[47] Petersen R.C., Smith G.E., Waring S.C., Ivnik R.J., Tangalos E.G., Kokmen E. (1999). Mild Cognitive Impairment: Clinical Characterization and Outcome. Arch. Neurol..

[48] Cotelli M., Calabria M., Manenti R., Rosini S., Maioli C., Zanetti O., Miniussi C. (2012). Brain Stimulation Improves Associative Memory in an Individual with Amnestic Mild Cognitive Impairment. Neurocase.

[49] Eliasova I., Anderkova L., Marecek R., Rektorova I. (2014). Non-Invasive Brain Stimulation of the Right Inferior Frontal Gyrus May Improve Attention in Early Alzheimer’s Disease: A Pilot Study. J. Neurol. Sci..

[50] Drumond Marra H.L., Myczkowski M.L., Maia Memória C., Arnaut D., Leite Ribeiro P., Sardinha Mansur C.G., Lancelote Alberto R., Boura Bellini B., Alves Fernandes da Silva A., Tortella G. (2015). Transcranial Magnetic Stimulation to Address Mild Cognitive Impairment in the Elderly: A Randomized Controlled Study. Behav. Neurol..

[51] Padala P.R., Padala K.P., Lensing S.Y., Jackson A.N., Hunter C.R., Parkes C.M., Dennis R.A., Bopp M.M., Caceda R., Mennemeier M.S. (2018). Repetitive Transcranial Magnetic Stimulation for Apathy in Mild Cognitive Impairment: A Double-Blind, Randomized, Sham-Controlled, Cross-over Pilot Study. Psychiatry Res..

[52] Ruthirakuhan M.T., Herrmann N., Abraham E.H., Chan S., Lanctôt K.L. (2018). Pharmacological Interventions for Apathy in Alzheimer’s Disease. Cochrane Database Syst. Rev..

[53] Cui H., Ren R., Lin G., Zou Y., Jiang L., Wei Z., Li C., Wang G. (2019). Repetitive Transcranial Magnetic Stimulation Induced Hypoconnectivity Within the Default Mode Network Yields Cognitive Improvements in Amnestic Mild Cognitive Impairment: A Randomized Controlled Study. J. Alzheimer’s Dis..

[54] Raichle M.E. (2015). The Brain’s Default Mode Network. Annu. Rev. Neurosci..

[55] Kim S.H., Han H.J., Ahn H.M., Kim S.A., Kim S.E. (2012). Effects of Five Daily High-Frequency RTMS on Stroop Task Performance in Aging Individuals. Neurosci. Res..

[56] Cohen R.A., Marsiske M.M., Smith G.E. (2019). Neuropsychology of Aging. Handb. Clin.Neurol..

[57] Solé-Padullés C., Bartrés-Faz D., Junqué C., Clemente I.C., Molinuevo J.L., Bargalló N., Sánchez-Aldeguer J., Bosch B., Falcón C., Valls-Solé J. (2006). Repetitive Transcranial Magnetic Stimulation Effects on Brain Function and Cognition among Elders with Memory Dysfunction. A Randomized Sham-Controlled Study. Cereb. Cortex.

[58] Antczak J., Kowalska K., Klimkowicz-Mrowiec A., Wach B., Kasprzyk K., Banach M., Rzeźnicka-Brzegowy K., Kubica J., Słowik A. (2018). Repetitive Transcranial Magnetic Stimulation for the Treatment of Cognitive Impairment in Frontotemporal Dementia: An Open-Label Pilot Study. Neuropsychiatr. Dis. Treat..

[59] Di Stasio F., Suppa A., Berardelli A. (2018). Frontotemporal Dementia: A Neurophysiological Study. Aging.

[60] Bonner M.F., Ash S., Grossman M. (2010). The New Classification of Primary Progressive Aphasia into Semantic, Logopenic, or Nonfluent/Agrammatic Variants. Curr. Neurol. Neurosci. Rep..

[61] Finocchiaro C., Maimone M., Brighina F., Piccoli T., Giglia G., Fierro B. (2006). A Case Study of Primary Progressive Aphasia: Improvement on Verbs after RTMS Treatment. Neurocase.

[62] Trebbastoni A., Raccah R., de Lena C., Zangen A., Inghilleri M. (2013). Repetitive Deep Transcranial Magnetic Stimulation Improves Verbal Fluency and Written Language in a Patient with Primary Progressive Aphasia-Logopenic Variant (LPPA). Brain Stimul..

[63] Benussi A., Dell’Era V., Cosseddu M., Cantoni V., Cotelli M.S., Cotelli M., Manenti R., Benussi L., Brattini C., Alberici A. (2020). Transcranial Stimulation in Frontotemporal Dementia: A Randomized, Double-blind, Sham-controlled Trial. Alzheimers Dement..

[64] Zhang X.-Q., Li L., Huo J.-T., Cheng M., Li L.-H. (2018). Effects of Repetitive Transcranial Magnetic Stimulation on Cognitive Function and Cholinergic Activity in the Rat Hippocampus after Vascular Dementia. Neural Regen Res..

[65] Veitia J.F.P. (2019). Repetitive Transcranial Magnetic Stimulation in the Rehabilitation of Vascular Dementia. Report of 2 Cases. Biomed. J. Sci. Tech. Res..

[66] León Ruiz M., Rodríguez Sarasa M.L., Sanjuán Rodríguez L., Benito-León J., García-Albea Ristol E., Arce Arce S. (2018). Current Evidence on Transcranial Magnetic Stimulation and Its Potential Usefulness in Post-Stroke Neurorehabilitation: Opening New Doors to the Treatment of Cerebrovascular Disease. Neurologia.

[67] Wang F., Geng X., Tao H.-Y., Cheng Y. (2009). The Restoration After Repetitive Transcranial Magnetic Stimulation Treatment on Cognitive Ability of Vascular Dementia Rats and Its Impacts on Synaptic Plasticity in Hippocampal CA1 Area. J. Mol. Neurosci..

[68] Rektorova I., Megova S., Bares M., Rektor I. (2005). Cognitive Functioning after Repetitive Transcranial Magnetic Stimulation in Patients with Cerebrovascular Disease without Dementia: A Pilot Study of Seven Patients. J.Neurol. Sci..

[69] Sedlackova S., Rektorova I., Fanfrdlova Z., Rektor I. (2008). Neurocognitive Effects of Repetitive Transcranial Magnetic Stimulation in Patients with Cerebrovascular Disease without Dementia. J. Psychophysiol..

[70] Morrin H., Fang T., Servant D., Aarsland D., Rajkumar A.P. (2018). Systematic Review of the Efficacy of Non-Pharmacological Interventions in People with Lewy Body Dementia. Int. Psychogeriatr..

[71] Liang X., Liu K., Guo L. (2010). Repetitive Transcranial Magnetic Stimulation (RTMS): A Possible Novel Therapeutic Approach to Dementia with Lewy Bodies. Med. Hypotheses.

[72] Jiang Y., Guo Z., McClure M.A., He L., Mu Q. (2020). Effect of RTMS on Parkinson’s Cognitive Function: A Systematic Review and Meta-Analysis. BMC Neurol..

[73] Lefaucheur J.-P., Aleman A., Baeken C., Benninger D.H., Brunelin J., Di Lazzaro V., Filipović S.R., Grefkes C., Hasan A., Hummel F.C. (2020). Evidence-Based Guidelines on the Therapeutic Use of Repetitive Transcranial Magnetic Stimulation (RTMS): An Update (2014–2018). Clin. Neurophysiol..

[74] Takahashi S., Mizukami K., Yasuno F., Asada T. (2009). Depression Associated with Dementia with Lewy Bodies (DLB) and the Effect of Somatotherapy. Psychogeriatrics.

[75] Khedr E.M., Mohamed K.O., Ali A.M., Hasan A.M. (2020). The Effect of Repetitive Transcranial Magnetic Stimulation on Cognitive Impairment in Parkinson’s Disease with Dementia: Pilot Study. Restor Neurol. Neurosci..

[76] Bhattacharjee S., Kashyap R., Abualait T., Annabel Chen S.-H., Yoo W.-K., Bashir S. (2021). The Role of Primary Motor Cortex: More Than Movement Execution. J. Mot. Behav..

[77] Macdonald R., Barnes K., Hastings C., Mortiboys H. (2018). Mitochondrial Abnormalities in Parkinson’s Disease and Alzheimer’s Disease: Can Mitochondria Be Targeted Therapeutically?. Biochem. Soc. Trans..

[78] Mammana S., Fagone P., Cavalli E., Basile M.S., Petralia M.C., Nicoletti F., Bramanti P., Mazzon E. (2018). The Role of Macrophages in Neuroinflammatory and Neurodegenerative Pathways of Alzheimer’s Disease, Amyotrophic Lateral Sclerosis, and Multiple Sclerosis: Pathogenetic Cellular Effectors and Potential Therapeutic Targets. Int. J. Mol. Sci..

[79] Andravizou A., Siokas V., Artemiadis A., Bakirtzis C., Aloizou A.-M., Grigoriadis N., Kosmidis M.H., Nasios G., Messinis L., Hadjigeorgiou G. (2020). Clinically Reliable Cognitive Decline in Relapsing Remitting Multiple Sclerosis: Is It the Tip of the Iceberg?. Neurol. Res..

[80] Leung I.H.K., Walton C.C., Hallock H., Lewis S.J.G., Valenzuela M., Lampit A. (2015). Cognitive Training in Parkinson Disease: A Systematic Review and Meta-Analysis. Neurology.

[81] Goel V. (2019). Hemispheric Asymmetry in the Prefrontal Cortex for Complex Cognition. Handb. Clin. Neurol..

[82] Kodounis M., Liampas I.N., Constantinidis T.S., Siokas V., Mentis A.-F.A., Aloizou A.-M., Xiromerisiou G., Zintzaras E., Hadjigeorgiou G.M., Dardiotis E. (2020). Assessment of the Reporting Quality of Double-Blind RCTs for Ischemic Stroke Based on the CONSORT Statement. J. Neurol. Sci..

[83] Rikos D., Dardiotis E., Aloizou A.-M., Siokas V., Zintzaras E., Hadjigeorgiou G.M. (2019). Reporting Quality of Randomized Controlled Trials in Restless Legs Syndrome Based on the CONSORT Statement. Tremor Other Hyperkinet. Mov..

